# Lord Salisbury's Address

**Published:** 1894-08-18

**Authors:** 


The Hospital

An Institutional Journal of

The Medical Sciences and Hospital Administration, with

An Extra Nursing Supplement.

Vol. XVI.—No. 412.] [Aug. 18, 1894.

### Lord Salisbury's Address.

When we recall the scoffs which were levelled at Boyle by the gentlemen and philosophers of Oxford in respect of his base and mechanical pursuits, we have some measure of the vast revolution of thought which has taken place between that day and our own, when at the meeting of the British Association in Oxford itself the chair of the President is occupied by one who is not only a leading statesman and an eminent thinker not unknown within the precincts of scientific investigation, but, moreover, Chancellor of the University and a great noble. The President's quotation from Keble indicates even more remarkably that this revolution is almost of yesterday, a testimony of no little strength to the power and penetration, as well as to the prevalent fashion of scientific thought.

In his address Lord Salisbury took up the character of *advocatus diaboli;* malicious observers may say, with some enjoyment. However this may be it is unwholesome for us to listen for ever to laudation of ourselves and to untempered appreciations of our own achievements. No doubt much of the repulsion once felt in Oxford and in the world from the new studies was due to a conflict between the old sciences of contemplation and the new sciences of experiment, but in our opinion the President did not sufficiently estimate repulsions of a far deeper kind—the fear of a new organon which would bring, not religion only, but, what was worse, social customs also to a relentless analysis. Lord Salisbury says, "Few men are now influenced by the strange idea that questions of religious belief depend upon the issues of physical research." If this be so, many men must be marvellously blind. Men have found, no doubt, that such changes come gradually, and they have learnt to regard them with more equanimity, but to allege that religious and social creeds have not been profoundly changed by the advances of positive knowledge since Keble's day is to close one's eyes.

Picturing, as he did in an eloquent figure, the lamp of science shedding a few pale rays upon a vast realm of darkness, and with his imagination impressed rather by the grandeur of the gloom than by the promise of the tiny light, the President, gracefully but needlessly calling himself a layman in the ranks of science, proceeded to delineate with a masterly hand the depths of the mystery and the ranks of the clouds. In no layman's fashion he went to the centre of the problem of the elements, showed the meaning of the discoveries of Mendeléef, and dealt with that hypothetical substance the ether after a fashion which showed his knowledge not to be but of yesterday. To say that the achievements of Dalton and Mendeléef, of Maxwell and Herz, do but thicken the mystery is as though one should say that an imperfect map makes one more ignorant of an unexplored continent. That it may impress us the more with the extent of the untravelled land may be true, but to speak of knowledge remaining as dark as ever betrays rather a hunger for ontological certainties than a satisfaction with the gradual tracking out of the order of nature.

Again for a moment Lord Salisbury seems to forget the scientific habit of mind, when, passing out of the circle of chemistry and physics, in which he is versed, he discusses the "mystery of life.' Is it not unscientific and therefore false, to speak of life as "a mysterious impulse which is able to strike across the ordinary laws of matter, and twist them for a moment from their path?" Is it not a scholastic notion and contrary to the conceptions we have painfully reached during this century, to speak of "laws of nature" as agents compelling matter from without? Life comes of the primary properties of matter as surely as do chemistry or physics, which latter in their several planes make for life and not against it; and we are at a loss to know what is meant by "*the* great *central* mystery" of life which we cannot penetrate, unless by it be signified some metaphysical entity; in this sense the central complexities of organization are no more mysterious than the animation of a black beetle.

After a fine tribute to the life and work of Darwin, the President ably arrayed in order two objections which he thinks are now unanswerable—the apparent conflict between the time required by the biologists and the time admitted by the astronomer and the geologist, and again the hypotheses of Weismann.

As regards the former objection, it cannot be alleged that species, taken altogether, "have not varied for 3,000 years." What measure is there for such a general assertion? Secondly, we cannot assume that all variations are "minute." Thirdly, we must remember that with the advance of time the relations between life and its media have become more complete and more stable. In earlier times when the arms of nature were more widely open adaptations may have been much more rapid. Finally, cosmic speculations are no less provisional than the biological: neither can pretend to control the other for some time to come.

To set the brilliant and stimulating but nebulous hypotheses of Weismann against Darwin's solid structure seems to indicate some lack of the sense of proportion.

The appeal to the law of chances in respect of the congress of the very pairs of animals who might generate successful varieties, whatever its applicability, must not be applied in forgetfulness of the facts which show that variation so far from being rare and by minute differences is very common, and by large differences. The waste of variation is probably very large indeed.

Even the noble and learned author of this masterly address shall not persuade us that the "acceptance of mere conjecture in the name and place of knowledge" is "the great danger of our time"; it has been a great danger in all times, but perhaps never less great than in our own day. Conjecture is none the less conjecture if it be old and respectable.

It were a poor compliment to Lord Salisbury's powerful criticism of positive science to treat it with insincere adulation.

